# The Study of Man

**Published:** 1895-03-02

**Authors:** 


### THE STUDY OF MAN.

WE are glad to welcome a fresh book on the study of anthropology,* especially since it is the first of a series under the editorship of Professor Starr, of Chicago, which is to deal with the kindred studies of ethnology, ethnography, prehistoric archæology, &c., and will supply a new library for popular reading on these comparatively modern sciences. Professor Mason, as curator of the Ethnological Department of the United States National Museum, has a claim to our attention on the subject. He divides his work into chapters under various headings: Woman the Weaver, the Skin-Dresser, the Beast of Burden, the Founder of Society, the Jack-of-all-Trades, and endeavours to prove to what a great extent woman has aided in the development of mankind with respect to industries, language, and religion. He stands up as a very Goliath in his championship of the fairer sex, and protests against the insinuation that woman had no part in making civilisation what it is. Yet we cannot help thinking that he overstates her case, or at any rate states it somewhat one-sidedly. The reader feels tempted to ask himself what the male portion of the community was doing all the time. Nevertheless, the author succeeds in causing us to reflect that women were by no means nonentities in the early days of civilisation. His conclusions are almost wholly drawn from modern observations of industries amongst less civilised communities, such as the Eskimo and Pueblos. The book contains a very large amount of information, which is somewhat scrappily put together, and its conclusions are, perhaps, rather hastily drawn. In the introduction the author gives the key to his plan, which is still further and better amplified in the concluding chapter, part of which might have been with advantage placed in the prelude. Herbert Spencer, the author tells us, divides the life-history of civilisation into the age of militancy and the age of industrialism. Professor Mason considers that these are rather sexual divisions—that there is a sex of militancy, and a sex of industrialism. This he parallels from our own age. Woman, he says, laid down the lines of her duties, *i.e.*, of industrialism, at the very beginning of human existence, and has stuck to them unremittingly. This was partly, perhaps, owing to physical inferiority to man, and partly, no doubt, to the cares of maternity, which would develop a tendency to stay at home.

Much has been related from time to time as to the cruel way in which the women are treated in savage communities, but this, Professor Mason tells us, is wholly erroneous, since such a policy would be suicidal. If the woman is cruelly treated, her power of bearing and properly nourishing children is impaired, and this would lead to the gradual extinction of the race.

Let us give a brief *résumé* of the book under its different heads. The first subject is woman as a food-bringer. Since the men of the family are engaged in hunting and fighting, or in fashioning their weapons, we are led to suppose that it devolves upon the women to seek food. Thus the latter are primarily responsible for the cultivation of herbs, for the manufacture of vessels of stone, of wicker, and of clay, and of the knives to aid her in the culinary department; also of granaries, and for the domestication of the cat to aid her in the defence of her hard-won supplies from the mice. She, too, provided the drink for the household, and invented pitchers and bottles. But it hardly seems sufficient proof of the *invention* of the plough, grindstone, and granary by woman merely to know that among some savage or primitive races of the present day women are the *users* of them. That women were the first weavers is not improbable; but to trace from the patterns of weaving a knowledge of geometry seems to be somewhat far-fetched. It is an interesting study, and one that is touched upon cursorily, to observe the connection between possible resources and products in the form of basket-work and of pottery, and from this we come to a discussion of the evolution of patterns, which runs parallel to the arguments already noticed in these pages.

The chapter on the skin-dresser naturally refers largely to the northern primitive folk of our own times, where this art is largely in the hands of the woman. Advantage is taken of this employment to attribute to woman the invention and manufacture of various kinds of scrapers. The next chapter, on pottery, is one of great interest, and links on closely to that of weaving; but perhaps it is of more value as a record of the art itself than of the inventive genius of the female sex. Types of pottery are of great importance in tracing the spread of particular clans and their influence on one another; and the kind of clay affords a means of ascertaining in some degree whether the pottery is local or imported.

The woman as a linguist supplies us with much food for reflection, and her influence on language is perhaps greater than might be supposed. The words for household utensils are likely to have been her invention, and further, it is true that the stay-at-home woman gossips over her work, and so has more need of words than the solitary man in pursuit of game. Again, since the woman teaches the children in their earliest years, it is she who gives them the most of their vocabulary. In this chapter Professor Mason seems to imply a variety of original starts for language, which would imply polygeny of the human race; a question which some of the greatest authorities still leave unsettled. Furthermore, women are the disseminators of language, through intermarriage, and capture by other tribes.

The remaining chapters of the book, though they contain much interesting information, do not, we think, prove woman to have had by any means a preponderant influence in the culture of the world. Society and clan life do not rest entirely on woman, nor does she play an important part in the evolution of religion. In the evolution of civilisation, in fact, it is impossible to draw any great distinction between the parts played by woman and by man. Each has a share in their own department; and the share of each is as important and as indissoluble in the progress as in the continuance of the race.

* "Woman's Share in Primitive Culture." By Professor O. T. Mason (Macmillan, 1895).

